# The Role of Endothelial–Mesenchymal Transition (EndMT) in Aneurysm Formation

**DOI:** 10.3390/biomedicines14081730

**Published:** 2026-07-31

**Authors:** Christoph Lange, Elena Kaschina

**Affiliations:** 1 Clinic for Anesthesiology and Intensive Care Medicine, Universitätsklinikum Brandenburg an der Havel, Hochstrasse 29, 14770 Brandenburg, Germany; c.lange@uk-brandenburg.de; 2 Institute of Pharmacology, Max Rubner Center for Cardiovascular Metabolic Renal Research (MRC), Charité—Universitätsmedizin Berlin, Chariteplatz 1, 10117 Berlin, Germany; 3 DZHK (German Centre for Cardiovascular Research), Partner Site Berlin, 10785 Berlin, Germany

**Keywords:** aneurysm, endothelial cells, endothelial–mesenchymal transition (EndMT)

## Abstract

Aneurysms represent a life-threatening disease due to their risk of rupture. Recent research points towards a role of endothelial–mesenchymal transition (EndMT) in their pathophysiology. EndMT acts as a cellular program during which endothelial cells undergo phenotypic and functional transformation into cells with mesenchymal properties. EndMT is implicated in embryogenesis, tissue repair, remodeling, angiogenesis and fibrosis. The studies performed in the last decade have gained new insights in the cell signaling pathways during aneurysm development in the context of EndMT and suggested new drug targets to prevent aneurysmal growth. This review summarizes the current knowledge on EndMT in various forms of aneurysm, such as abdominal and thoracic aortic aneurysms, intracranial aneurysms and coronary artery aneurysm due to Kawasaki disease.

## 1. Introduction

Endothelial–mesenchymal transition (EndMT) is a cellular reprogramming process by which endothelial cells lose their specific phenotype and transform into mesenchymal cells [1]. Besides its crucial role in cardiac embryogenesis [2] and tumor growth, EndMT is of importance in cardiovascular diseases [3,4,5]. In particular, EndMT contributes to atherosclerosis [6,7], pulmonary arterial hypertension [8], diabetic vasculopathy [9], cardiac fibrosis [10], mitral valve prolapse [11] and vascular occlusion as well as neointima formation [12].

In the last decade, increasing evidence has suggested the role of endothelium dysfunction and EndMT in the formation and progression of aneurysms. Endothelial cell (EC) dysfunction might promote monocyte recruitment and inflammatory signaling cascade and raise vascular damage, resulting in extracellular matrix degradation, disruption of elastic fibers, and aneurysm progression and rupture [13]. EC dysfunction is closely connected to EndMT, a pathophysiological process that has recently gained great attention and inspired new research.

In response to different stimuli, for example IL-1β, TGF-β, TNF-α, disturbed blood flow, oxidative stress, and dysregulation of Notch and Wnt signaling, ECs differentiate towards mesenchymal-like cells which gain proliferative, migratory and invasive properties. These cells are characterized by increased cytoskeletal and matrix proteins that are typical for fibroblasts and myofibroblasts [14]. They release proteases and activate neutrophils and macrophages, contributing to the inflammation, damage, remodeling and fibrotic transformation of the vascular wall [15].

Mesenchymal-like cells diminish their endothelial-specific markers such as PECAM-1/CD3, VE-cadherin/CDH5, vWF and endothelial cell adhesion molecules, including tight junction proteins ZO-1 and Claudin-1. The mesenchymal-like cells consequently express mesenchymal markers that comprise for example TAGLN/SM22a, α-SMA/ACTA2, FSP-1/S100A4, fibrillary collagens type I and type III (COL1A1, COL3) and several others (A1CD105, SCA1, fascin1, HSP47, etc.) depending on the tissues and stimuli [4].

EndMT is also orchestrated by transcription factors such as Snai1, SLUG (Snai2), Twist, ZEB1/2 and β-catenin, which repress endothelial genes and activate mesenchymal programs [4].

Epigenetic modifiers such as DNA methyltransferases, HDACs, and histone methyltransferases as well as post-transcriptional mechanisms like microRNA have also been linked to EndMT control [16].

On the other hand, several factors can suppress EndMT, for example normal laminar shear stress, BMP-7 (Bone Morphogenetic Protein 7) and FGF (Fibroblast Growth Factor), endothelial fatty acid oxidation and high-density lipoproteins (HDLs) [17]. By targeting different signaling pathways (TGF-β, Notch, Wnt/β-Catenin, PPAR-γ, PDGF, DPP-4), epigenetic and post-transcriptional mechanisms can also partially reverse EndMT [1,16,18].

Endothelial dysfunction, EndMT and their major biochemical signaling pathways during atherosclerosis and cardiovascular disease progression have been thoroughly reviewed recently [1,3,4,6]. Atherosclerosis and aneurysms often coincide, and their risk factors and pathogenesis entail commonalities [4,19]. Nevertheless, the changes in the wall structure in atherosclerosis do not usually lead to weakening of the vessels and outward remodeling, whereas vessel dilatation by aneurysms is caused by the loss of elastic fibers, structural integrity and tensile strength [14]. Thus, over the last decade research attention has been paid to the role of EndMT in vascular remodeling during aneurysm formation.

In this mini-review, we analyze and summarize recent experimental and human studies on the involvement of EndMT in aneurysm development, we focus on the advancements in the study of EndMT in different forms of aneurysm disease, and finally, we discuss the importance of EndMT as a therapeutic target in aneurysms.

## 2. Endothelial Dysfunction, EndMT and Aneurysms

An aneurysm is an irreversible dilatation of the blood vessel due to wall weakness. Aneurysms have different development origins, genetic and behavioral risk factors, flow patterns and structural differences in the vessel wall [14]. Nevertheless, aneurysms are characterized by common histological features such as medial degeneration with the loss of vascular smooth muscle cells, destruction of elastic fibers and adventitial inflammatory cell infiltration. The key mechanisms of aneurysm development include chronic inflammation, oxidative stress, intraluminal thrombus formation, neovascularization, increased proteolysis and extracellular matrix degradation [20]. These mechanisms partly overlap with the pathogenesis of atherosclerosis, particularly in abdominal and intracranial aneurysms [4,19].

There is increasing evidence that endothelial dysfunction is an early event both in atherosclerosis and in aneurysm formation [21]. It promotes monocyte recruitment and an inflammatory signaling cascade and also contributes to oxidative stress via endothelial nitric oxide synthase (eNOS) uncoupling [21]. Endothelial dysfunction is considered a potentially reversible event, whereas EndMT contributes to irreversible structural remodeling, fibrosis, and extracellular matrix disorganization, ultimately promoting vessel wall instability and dilation [1].

Interestingly, ECs exhibit marked heterogeneity in gene expression and reactivity across different vascular beds [22,23]. In response to inflammatory stimuli, ECs also display sex-specific differences that are non-dependent on hormone regulation [24]. Furthermore, there are variations in the susceptibility of EC to undergo EndMT depending on vessel localization [25]. Thus, it could be expected that EndMT processes are non-identical in different vascular beds and, hence, in different forms of aneurysm. Here, we summarize and discuss the recent discoveries on abdominal, thoracic, intracranial and coronary artery aneurysms in Kawasaki disease in context of EndMT processes.

### 2.1. Abdominal Aortic Aneurysm (AAA)

Our group proposed the role of EndMT in AAA development while studying the impact of the Ang II receptor type 2 (AT2) receptor in a rat model of inflammatory, elastase-perfused aneurysm [26,27]. The induction of aneurysm provoked an upregulation of TGF-β1, which is known to initiate EndMT [8]. Indeed, transcriptional factors SLUG and Snai1 were expressed in the adventitia of aorta, pointing to the possible origin of the EndMT process from the endothelium of the *vasa vasorum*. The expression of these factors coincided with the triggering of the necroptosis factor pseudokinase mixed lineage kinase domain-like (MLKL) as well as with matrix metalloproteinases MMP-2 and MMP-9. Moreover, treatment with the Ang II receptor type 2 agonist C21 ameliorated aortic dilatation, inflammation, proteolysis, cytokine and TGF-β1 expression, and, additionally, positively influenced the EndMT marker SLUG. Although EndMT was not the primary aim of this investigation, first insights into the role of this pathological process in experimental AAA have been provided.

Recently, Millar and co-authors (2024) gained deeper understanding of EndMT during aortic aneurysm formation in mice by using a modified elastase AAA model with the periadventitial application of elastase. Aneurysmatic aortic tissues were characterized by increased expression of typical EndMT transcription factors Snai1, SLUG, Twist and ZNF [28]. The loss of endothelial cell markers VE-cadherin, eNOS and vWF and overexpression of mesenchymal cell markers S100 and α-SMA confirmed stimulation of EndMT. Given that these changes could be attenuated by IL-1 receptor knockout and pharmacologic inhibition of the IL-1 receptor with anakinra, the authors suggested an interaction between EndMT, inflammation and endothelial dysfunction [28].

Weng et al. (2025) added further evidence on the role of EndMT in Ang II-induced AAA in ApoE−/− mice, a widely known model of atherosclerotic aneurysm [29]. During aneurysm progression, the endothelial cell markers PECAM1, CDH5, KDR and COL4A1 were downregulated, indicating a decline in endothelial cells. Concomitantly, EndMT marker genes Vim, Acta2, Col1a1, and Fn1 and EndMT-associated transcription factors Twist1, Twist2, Snai1 and Zeb2 were upregulated. Further, the authors proposed the regulation of EndMT by a member of the TGF-β superfamily, bone morphogenetic protein 4 (BMP4). As shown in endothelial cells, BMP4 induced phenotypic transition, inflammation, and cell migration and invasion via the PI3K/AKT/mTOR pathways. In consequence, the inhibition of BMP4 prevented EndMT and inflammation in aneurysm.

Another important study was performed by Wang J.C. et al. (2025) in Ang II-infused mice [30]. In the aneurysmatic tissues of mice, the authors found highly expressed Snai1, α-SMA, phospho-extracellular signal-regulated kinase (p-ERK1/2) and MMP-2 alongside low levels of endothelial markers CD31 and VE-cadherin. Notably, silencing of Snai1 expression correlated with suppression of EndMT and decreased AAA incidence and severity, pointing to the crucial role of EndMT in this AAA model. The authors also proposed that Snai1 expression, in turn, is modulated by miRNA-325 because its administration decreased Snai1 and MMP-2 in aorta and ameliorated AAA growth.

The cellular heterogeneity and transcriptional signatures of ECs have been revealed by Wu et al. (2025) in human and mouse abdominal aortas using single-cell RNA sequencing (scRNA-seq) technology [31]. In aortas from Ang II and salt-induced AAA mice, the authors identified four subpopulations of ECs: lipid-handling, Fn1+ mesenchymal-like, pleiotropically activated, and lymphatic-like. Moreover, the authors revealed the role of transcription factor Sox18 in AAA, because its overexpression in ECs decreased EndMT and prevented AAA development. This protective effect of Sox18 was attributed to the regulation of the PI3K/Akt signaling pathway.

A modified model of AAA was studied in ApoE−/− mice with partial suprarenal ligation of the aorta [32]. This experimental model enabled the researchers to uncover the impact of disturbed flow (d-flow) in EndMT induction and progression of AAA. Blockade of the TGF-β/Smad signaling pathway with Galectin-7 resulted in the inhibition of EndMT in the cells and further reduced the aneurysm diameter and decreased the mortality of AAA mice. Altogether, this work provided direct evidence for the critical role of EndMT in AAA, particularly in the context of disturbed flow and the TGF-β/Smad signaling pathway.

Human studies describing EndMT in AAA are scarce and restricted to the comparison of the marker’s expression in aneurysmatic and non-aneurysmatic tissues. So far, only two studies have shown the regulation of EndMT markers in comparison to healthy aorta. For example, tissues from human AAA showed an upregulation of SLUG and an increased staining of the mesenchymal cell marker S100 [28]. In endothelial lysates from human AAA segments, expression of endothelial markers occludin, CD31, and ZO-1 was markedly reduced compared with adjacent non-aneurysmal aorta, whereas mesenchymal markers SM22α, α-SMA, Vimentin and Fibronectin were significantly upregulated. The authors explained such regulation by a d-flow-driven EndMT in the aneurysmal endothelium that is consistent with previously mentioned animal experiments [32].

In summary, evidence for the involvement of EndMT in the pathogenesis of AAA has been provided in various experimental models, both inflammatory and atherogenic, as well as in human studies on EndMT marker expression. The prevention of the EndMT pathways by inhibiting its key activators, especially IL-1 and the TGF-β/Smad signaling pathway, may be beneficial in AAA. The endothelial source of EndMT besides aortic intima could be the *vasa vasorum* vessels of the adventitia.

### 2.2. Thoracic Aortic Aneurysm (TAA)

The role of endothelial cells in the pathogenesis of thoracic aortic aneurysm (TAA) and dissection (TAAD) has been newly summarized by Verstraeten and coauthors (2023) [33]. Several endothelial mechanisms have been suggested to be involved in TAAD development, i.e., dysregulation of endothelial nitric oxide (NO), oxidative stress-driven endothelial dysfunction, dysregulation of Ang II or TGF-β signaling and genetic mechanisms. Gould and coauthors (2019) first provided genetic evidence for endothelial dysfunction and EndMT as causal in TAA [34]. The authors identified pathogenic variants in the ROBO4 gene, encoding a transmembrane receptor in patients with bicuspid aortic valve and ascending aortic aneurysm (BAV/AscAA). Targeted silencing of this gene or mutant overexpression in endothelial cells resulted in impaired barrier function and a synthetic cell molecular signature typical for EndMT. These findings were confirmed in patients and in ROBO4-deficient animal models.

Molecular mechanisms of aortopathy in bicuspid aortic valve (BAV) patients were studied by Maleki et al. (2016) [35]. Proteomics and molecular biologic analysis of the samples from dilated and non-dilated ascending aortas revealed a molecular signature of an endothelial/epithelial–mesenchymal transition-like process that could contribute to aortic instability and dilatation.

Accordingly, Millar et al. (2024) reported increased Snai1 and SLUG signaling within TAA tissues compared with controls but no difference in S100 and vWF expression [28].

Terriaca et al. (2023) shed light on the phenotypic switching of endothelial cells in Marfan syndrome TAA, describing Wnt/β-catenin activation and a decrease in endothelial marker C31 in aorta [36].

Another study coming from the same group provided evidence for miRNA dysregulation that drives specific EndMT patterns in different TAAs. For example, Marfan syndrome patients exhibit higher EndMT, driven by upregulated miR-632 [37]. Functional evidence shows that endothelial cells derived from dilated aortas of BAV have attenuated Notch signaling with impaired EndMT induction [38]. Unfortunately, there exist no data concerning EndMT in TAA from preclinical studies.

Summarizing, all these findings identify a novel endothelial etiology for TAA that requires further in-depth investigations especially in context of genetic mechanisms.

### 2.3. Intracranial Aneurysm (IA)

The pathological features of intracranial aneurysm (IA) share many similarities with atherosclerosis, such as high wall shear stress, endothelial dysfunction, inflammation, phenotypic and functional alterations of vascular smooth muscle cells, and extracellular matrix remodeling and disruption of the internal elastic lamina [39,40]. Although the importance of EndMT in atherosclerosis is well described [4], we generally have only sparse knowledge about EndMT in IA.

Jamous et al. (2007) first evaluated the in vivo mechanisms of IA in an experimental cerebral aneurysm model in rats with renal hypertension and right common carotid artery ligation [41]. By comparing vessel morphology with molecular pathological changes at different time points, the authors provided evidence that the formation of IA begins with endothelial injury at the apical intimal pad. This process was followed by inflammation and partial defect in the artery.

A further study performed in rats found that miR-29 expression and activation of the TGFBR2/Smad3 axis promote EndMT and induce the progression of IA [42].

Jabbarli et al. (2020) reviewed 123 human studies on histological and molecular markers assessed in human healthy, IA and ruptured samples [43]. After analyzing 350 markers and 20 associated functional pathways contributing to IA formation, the authors highlighted the role of endothelial layer damage and the endothelial dysfunction pathway. Of note, EndMT-associated diagnostic markers of IA such as TGF-β and SOX4 and the transcription factor Twist1, as well as IL-1β, NF-κB and MMP-2, were increased in IA.

A recent study by Jiang et al. (2022) was focused on human EndMT-related genes in healthy, ruptured and unruptured cerebral arteries [44]. Bioinformatics gene analysis revealed differentially expressed genes (*ADIPOQ*, *WNT11*, and *CCL21*) in all groups. Interestingly, two genes, Fibronectin 1 (*FN1*) and secreted protein acidic and cysteine-rich (*SPARC*), were differentially expressed in aneurysmatic and healthy vessels. These genetic findings correlate with previous results from Li et al. (2013) showing SPARC upregulation in IA, concurrently with MMP-2 and MMP-9 overexpression [45]. In addition, FN1 might also be a candidate gene target for further investigations because it is involved in the maintenance of the ECM of the arterial wall by human IA, as previously identified via SNP analysis [46].

Although endothelial dysfunction has been shown to contribute to the pathogenesis of IA, studies on the role of EndMT are still limited. Further research is needed to provide strong evidence for a link between EndMT and IA.

### 2.4. Coronary Artery Aneurysm in Kawasaki Disease

Kawasaki disease (KD) is an acute vasculitis in children that is characterized by inflammation of medium-sized muscular arteries and coronary artery aneurysms [47]. If not treated, the aneurysms progress rapidly, reaching maximum size by 6 weeks after illness onset. An immunologic response is mainly elicited in genetically susceptible children after exposure to a trigger. The main regulated cellular signaling pathways include IL-6, the TGF-β/T helper (Th)-17 axis and the IL-12/interferon gamma axis [47].

It has also been suggested that in the coronary arterial wall of KD patients, the TGF-β signaling mechanism is responsible for the generation of myofibroblasts which drive collagen network remodeling, antigen presentation, and recruitment of regulatory T-cells and pro-inflammatory neutrophils and macrophages [48].

He et al. (2017) assessed molecular markers of EndMT in acute KD by incubating sera from healthy children or those with KD with human umbilical vein endothelial cells (HUVECs) [49]. Sera from KD patients suppressed the Krüppel-like factor 4 (KLF4)-miR-483 axis in endothelial cells, leading to increased expression of connective tissue growth factor (CTGF) and activation of EndMT [49]. This mechanism may influence vascular wall injury and aneurysm formation in KD. Interestingly, statin therapy partly inhibited EndMT and restored endothelial metabolism, suggesting a benefit of statins in acute KD patients.

More recently, Ni et al. (2022) found a decrease in the endothelial marker ZO-1 and an upregulation of EndMT markers Twist, Snai1 and Vimentin in HUVECs exposed to serum from KD patients as well as in a mouse model of KD [50]. Moreover, the authors demonstrated that circular RNAs (circ3302) induce EndMT as evidenced by EndMT-related markers.

Collectively, these findings support the role of EndMT in coronary artery wall injury in KD. Particularly, the mesenchymal-like cells that secrete pro-inflammatory and matrix-remodeling factors may contribute to the formation of coronary artery aneurysm.

## 3. Discussion

**Figure 1:**
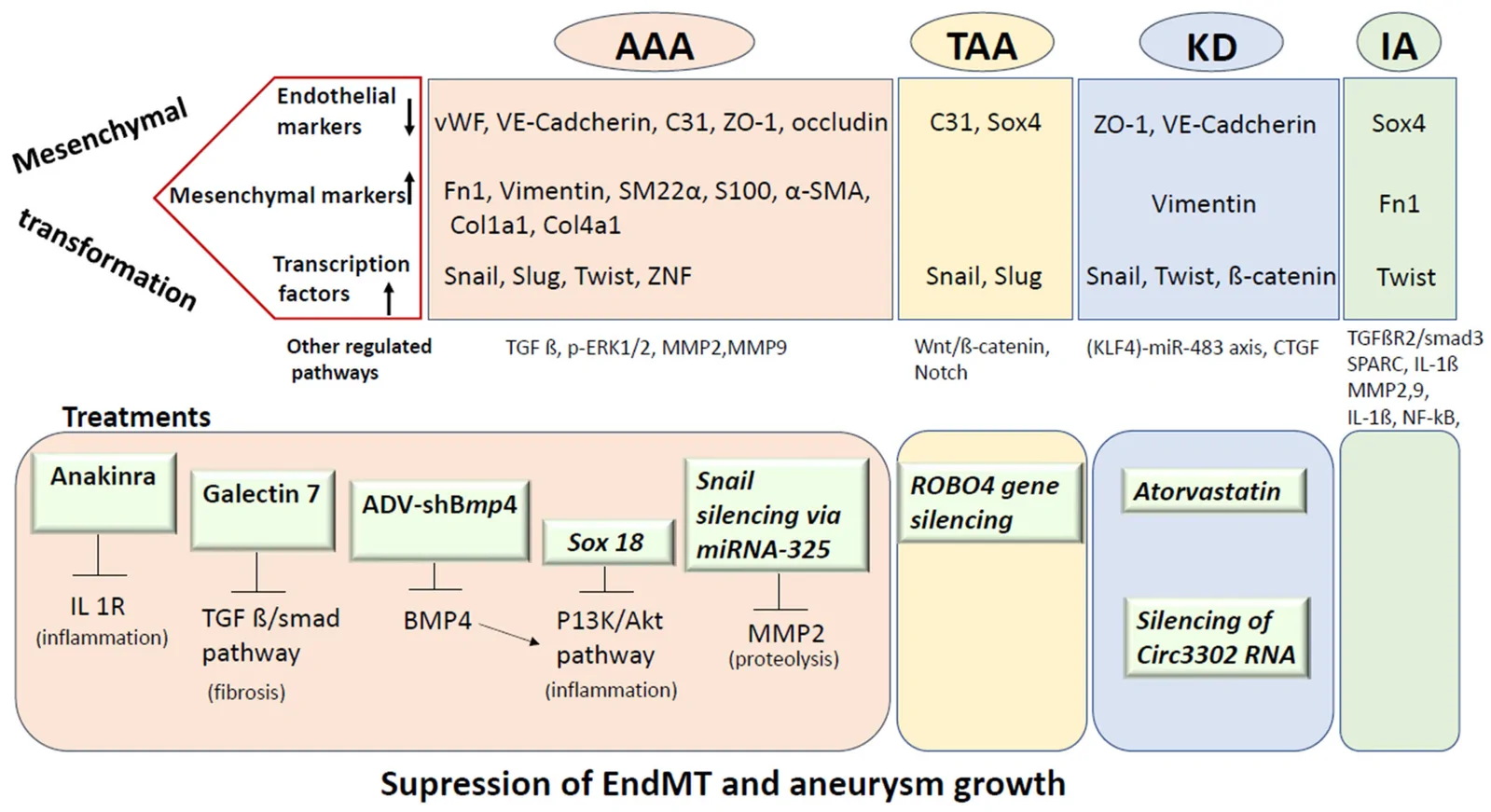
This scheme illustrates the endothelial–mesenchymal transition by various aneurysms. For detailed explanation, refer to the respective paragraph of the article. Abbreviations: EndMT—endothelial–mesenchymal transition; AAA—abdominal aortic aneurysm; TAA—thoracic aortic aneurysm; KD—Kawasaki disease; IA—intracranial aneurysm.

This review summarizes the current knowledge on EndMT in different types of aneurysms. The main results are presented in Figure 1 and more detailed information about the studies is summarized in Table 1. The aneurysms differ not only in their localization, but also in pathophysiological aspects including the temporal course of disease progression and the distinct pathomechanisms leading to aneurysm development.

The majority of animal and human studies have been performed in AAA. This type of aneurysm has received more research attention due to its high incidence and high mortality rate as a consequence of rupture [51]. The strongest predictor of AAA rupture is an increased aortic diameter. Age (>65), male gender, atherosclerosis and smoking are the main risk factors of AAA. The key features of AAA are chronic inflammation, oxidative stress, apoptosis of vascular smooth muscle cells and proteolytic fragmentation of the extracellular matrix [14]. These mechanisms contribute to mechanic instability, dilatation and finally to aortic rupture. Cytokines, for example IL-1β, TGF-β, IL-6, IL-8 and TNF-α, released from immune cells, activate downstream cell signaling; set in motion inflammation, proteolysis, remodeling, and fibrosis; and initiate EndMT as well. Interestingly, nicotine via its receptor α7nAChR activates ERK1/2 signaling, upregulates Snai1, downregulates endothelial markers VE-cadherin and CD31 and upregulates mesenchymal markers α-SMA and FSP1 [52]. Hence, smoking might additionally initiate the EndMT process in the vessels. Indeed, AAA often but not always coincides with atherosclerosis. In AAA, EndMT markers were regulated in different experimental models, both atherosclerotic [29,30,31] and non-atherosclerotic [26,28], providing supporting evidence for a direct role of EndMT in AAA. Through the time course of aneurysm formation, the number of endothelial cells declines, coinciding with increased expression of EndMT markers Vimentin, Acta2, Col1a1, and Fn1 and EndMT-associated transcription factors Twist1, Twist2, Snai1, and Zeb2 [29]. The EndMT process in the aorta might be initiated by the endothelial cells originating from the intima and/or *vasa vasorum* of the adventitia. This was confirmed histopathologically in different animal models with the application of elastase either in the aorta [26] or in the adventitia [28]. Importantly, human aneurysmal tissues also expressed more EndMT markers compared with non-aneurysmal aorta [28,32].

Current experimental studies have provided evidence for suppression of EndMT and aneurysmal growth by the antagonist of the IL1 receptor anakinra [28], blockade of the TGF-ß/Smad pathway by Galectin-7 [32], overexpression of transcription factor SOX18 that suppresses the P13K/Akt pathway [53] and Snail silencing via miRNA-325 [30]. The inhibition of BMP4, a member of the TGF-ß superfamily, prevented EndMT and inflammation in the aorta [29]. Altogether, these findings suggest that suppression of EndMT may provide protection against aneurysm remodeling, although further extensive functional studies are needed to confirm the benefits of these treatments. On the other hand, inhibition of EndMT may be potentially unsafe, because it participates in vascular repair and remodeling [1,3,4]. These studies also support a cross-talk between endothelial dysfunction, EndMT, inflammation, proteolysis and fibrosis by AAA (Figure 1). Whether EndMT plays a causal role in the protection or it is a consequence of the downstream signaling after inhibition of receptors like IL-1R or TGF-ß remains to be answered.

Despite previous intensive investigations of a wide range of drug classes, no medical therapy options have been found to be sufficient in preventing AAA. Research is currently focused on the antidiabetic drug metformin which has the potential to reduce AAA growth in humans by ameliorating extracellular matrix remodeling and inflammation [54]. Metformin prevented AAA formation in a rodent non-atherosclerotic model by activating the AMPK/mTOR signaling pathway [55]. Regarding EndMT regulation, metformin suppressed IL-1β and TGF-β1-induced EndMT through inhibition of SOX4 in endothelial cells [56].

Although the effects of statins on AAA have been discussed controversially, a recent population-based study has evidenced an association between the dose of statins and reduced AAA growth rate [57]. Furthermore, simvastatin inhibited induced EndMT in HUVECs by decreasing oxidative stress, suppressing TGF-β/Smad signaling and inactivating the transcription factors Snail-1 and Twist-1 [58]. Therefore, metformin and statins are promising drugs for AAA treatments, which might also interrelate with the EndMT process.

TAA is characterized by enlargement of the thoracic aorta, often with dissection occurring at young ages. Pathophysiological mechanisms include intrinsic defects in the elastin and collagen components of the aortic wall, medial degeneration and dissection [14]. The development of TAA is influenced by genetic predisposition, embryological factors, inherited defects in ECM and atherosclerosis and arterial hypertension. Special attention is paid to the role of dysregulation of canonical and noncanonical TGF-β signaling, which, for example, develops due to fibrillin-1 gene mutation by Marfan syndrome [59].

Regarding EndMT processes, Marfan patients exhibited higher EndMT, driven by upregulated miR-632 [36] and Wnt/ß catenin activation [36], whereas patients with bicuspid aortic valve disease exhibited attenuated Notch signaling [38]. A molecular signature of an endothelial/epithelial–mesenchymal transition-like process has also been described in human TAA [35]. EndMT transcriptional factors Snail, Slug, Twist and ß-catenin which repress endothelial genes and activate mesenchymal programs [4] were increased in TAA, like in the other types of aneurysms. It has been evidenced that EndMT alterations in TAA are attenuated via targeted silencing of the *ROBO4* gene [34]. This study has increased our understanding of genetic predisposition and abnormal endothelial function in the pathogenesis of TAA. The treatment via targeted gene silencing is promising and TAA-specific, but requires further confirmation in clinical trials.

In Kawasaki disease, an acute vasculitis in children under 5 years, the coronary aneurysm progresses rapidly within 6 weeks. This acute inflammatory condition is considered to be an immunologic response to infections accompanied by antigen presentation, recruitment of pro-inflammatory cells and activation of IL-6, TGF-ß/Th-17 and IL-12/interferon gamma pathways [47]. Hence, KD differs from aortic aneurysms in the pathogenesis and clinical aspects. Nevertheless, an initiation of EndMT by KD has been evidenced in experimental studies by using serum from patients with the disease [50]. Furthermore, suppression of the Krüppel-like factor 4 (KLF4)-miR-483 axis followed by activation of connective tissue growth factor (CTGF) stimulated the EndMT process, which could be attenuated by atorvastatin [49]. The anti-inflammatory effects of statins together with the effects on EndMT [58] might be beneficial in KD patients and realistic for clinical application. Silencing of Circ3302 RNA has been suggested as a novel option for EndMT inhibition [50].

Intracranial or cerebral aneurysm is a dilatation of a brain artery that has a prevalence of 3% in adult populations and its rupture risk increases with age. Besides well-known risk factors, such as hypertension and smoking, infection, trauma, female gender and genetic susceptibility play an important role in IA formation [39].

In IA, shear stress causes endothelial layer damage and, subsequently, an inflammatory response with the recruitment of inflammatory cells and activation of endothelial dysfunction followed by EndMT. Direct diagnostic EndMT markers Sox 4, Fn and Twist 1 as well as associated pathways TGF-ßR2/Smad3, SPARC, IL-1ß, MMP-2,9, IL-1ß, and NF-kB have been identified in cerebral aneurysms [42,43,44,45,46]. Moreover, a gene differential analysis of ruptured and unruptured IA groups suggested that angiogenesis and endothelial cell proliferation play important roles, possibly via the PI3K-AKT signaling pathway [44]. Indeed, all these findings point to a complex interplay between EndMT and other cellular pathomechanisms, but functional evidence for the regulation of the EndMT process in IA has still not been provided.

Overall, the described aneurysms are distinct in their origin, risk factors, temporal course of disease progression and pathomechanisms. Recent investigations suggest that EndMT contributes to the pathogenesis of all types of aneurysms, possibly like a basic mechanism of an endothelial injury response. Nevertheless, inhibition of EndMT by using different approaches points to aneurysm-type-specific regulation. Further extensive functional studies are needed to confirm the mechanisms of EndMT across different aneurysm forms.

## 4. Limitations of the Study

Despite the growing body of evidence implicating EndMT in aneurysm pathogenesis, several important limitations of the current literature should be acknowledged. First, most available evidence is derived from experimental animal models and in vitro studies, whereas investigations in human aneurysm tissue remain descriptive and limited. Consequently, the translational relevance of several proposed EndMT-associated pathways has yet to be validated in proof-of-concept studies.

Second, considerable heterogeneity exists among experimental models, including elastase-induced, angiotensin II-induced, disturbed-flow, and genetically modified models. These models reproduce distinct aspects of aneurysm biology and may therefore activate different EndMT states, limiting direct comparison between studies. Likewise, endothelial cells display pronounced vascular-bed-specific and disease-specific heterogeneity, suggesting that EndMT is unlikely to represent a uniform process across abdominal, thoracic, intracranial, and coronary aneurysms.

Finally, another important limitation is the lack of standardized criteria for defining EndMT. Most studies rely on the altered expression of endothelial and mesenchymal markers, while only a limited number employ lineage-tracing approaches, single-cell transcriptomics, or spatial transcriptomic analyses to directly demonstrate endothelial cell fate conversion. As many EndMT markers are not specific to endothelial-derived mesenchymal cells, distinguishing complete EndMT from partial EndMT or other forms of cellular plasticity remains challenging.

## 5. Conclusions and Outlook

A large number of experimental and clinical studies in the last decade evidenced the relevance of EndMT in the pathogenesis of aneurysm. The downregulation of endothelial markers and increased expression of mesenchymal markers and transcriptional factors related to EndMT confirmed the presence of this cellular reprogramming process in the vascular wall of aneurysms. Further studies should define special molecular signatures of EndMT in different types of aneurysms.

Progress has been made in unraveling the multifactorial nature of EndMT in aneurysm development. In particular, the TGF-β/Smad, Wnt/β-catenin, and IL-1 signaling pathways and epigenetic and post-transcriptional mechanisms like microRNA are shown to be relevant in the regulation of vascular remodeling in aneurysms. Moreover, the first genetic evidence (ROBO4 gene) for endothelial dysfunction and EndMT as causal in TAA has been provided.

Selective modulation of mesenchymal reprogramming might attenuate pathological remodeling and prevent the degradation of the extracellular matrix and rupture. Several treatment options like Snai1-silencing, microRNA-325, inhibition of TGF-β/Smad by galectin7 and BMP4, inhibition of IL-1R by anakinra, and overexpression of transcription factor SOX18 have recently been suggested.

Given that endovascular repair has become a standard treatment in aortic aneurysms, new findings in the context of EndMT could unravel the mechanism of aortic weakening after stent graft implantation. Furthermore, the *vasa vasorum* may represent an interesting research target, because these vessels provide a source of endothelial cells prone to EndMT.

Altogether, accumulating evidence supports an association between EndMT-like endothelial plasticity and aneurysm remodeling, but direct causal and translational evidence remains incomplete.

## Figures and Tables

**Table 1:** Experimental and human studies on EndMT in different types of aneurysm.

Type of Aneurysm	Model	EndMT Markers	Regulation	Intervention (Prevention of EndMT)	Authors
AAA	Rat, elastase perfusion	Snail, MLKL, MMP2,9 Slug	↑ ↓	AT2 Receptor agonist C21	[26]
AAA	CDH5-Cre eYFP+/+ mice periadventitial elastase application	Slug, Snail, Twist, ZNF281, S100, alpha SMA VE Cadherin, eNOS, vWF	↑ ↓	IL1R antagonist Anakinra	[28]
AAA	Human	Snail, Slug, s100 vWF	↑ ↓		[28]
AAA	ApoE−/− mice, Ang II-induced	Twist1, Twist2, Snail, Zeb2, Vim, Acta2, Col1a1, Fn1 Pecam, Cdh5, Kdr, Col4a1	↑ ↓	Inhibition of BMP4 via ADV-sh*Bmp4*	[29]
AAA	ApoE−/− mice, Ang II-induced	α-SMA, Snail p-ERK1/2, MMP-2 CD31, VE-cadherin	↑ ↓	SNAI1-Silencing MicroRNA-325	[30]
AAA	Ang II and salt-induced AAA, mice	Fn1+ mesenchymal-like cells	↑	Sox18	[31]
AAA	ApoE−/− mice, partial suprarenal ligation of aorta	Vim, Fn1 CD31, ZO1	↑ ↓	Galectin 7 (TGF-β/smad signaling Blocker)	[32]
AAA	Human	Vim, Fn1, SM22α, α-SMA Occludin, CD31, ZO1	↑ ↓		[32]
BAV/AscAA	ROBO4 deficient mice	*Robo4* knockout causes aortic valve defects and aortic aneurysm in mice			[34]
BAV/AscAA	Human	ROBO4+ cells in aneurysm α-SMA	+ ↑		[34]
TAA	Human	Snail, Slug	↑		[28]
Aortopathy in Bicuspid aortic valve (BAV) patients	Human	Molecular signature of an endothelial/epithelial–mesenchymal transition-like process			[35]
Marfan syndrome TAA	Human	C31 Wnt/β-catenin activation miR-632	↓ ↑ ↑		[36]
IA	Rat, carotid and renal artery ligation	miR-29, TGFBR2/Smad3	↑		[42]
IA	Human	SPARC, FN1	↑		[44]
IA	Human	SPARC, MMP2, MMP9	↑		[45]
IA	Human	FN1	↑		[46]
Kawasaki disease (KD)	Human, serum	CTGF (KLF4)-miR-483 axis	↑ ↓	Statin	[49]
KD	Human HUVECs exposed to serum from KD patients	Twist, Snail, Vimentin ZO-1	↑ ↓	Silencing of circ33-02	[50]
KD	Mice, candida albicans cell wall extract (CAWS) model	Twist, Snail, Vimentin, β-catenin VE-cadherin, ZO-1	↑ ↓		[50]

Abbreviations: EndMT—endothelial-mesenchymal transition; AAA—abdominal aortic aneurysm; BAV—bicuspid aortic valve; AscAA—ascending aortic aneurysm; TAA—thoracic aortic aneurysm; KD—Kawasaki disease.

## Data Availability

No new data were created or analyzed in this study. Data sharing is not applicable to this article.

## Abbreviations

The following abbreviations are used in this manuscript:

AAA	Abdominal Aortic Aneurysm
ACTA2	Actin Alpha 2, Smooth Muscle
ADIPOQ	Adiponectin
AngII	Angiotensin II
ApoE	Apolipoprotein E
AscAA	Ascending Aortic Aneurysm
AT2	Angiotensin II Type 2 Receptor
BAV	Bicuspid Aortic Valve
BMP4	Bone Morphogenetic Protein 4
BMP-7	Bone Morphogenetic Protein 7
C21	Selective Angiotensin II Type 2 Receptor Agonist
CCL21	C-C Motif Chemokine Ligand 21
CD31	Cluster of Differentiation 31 (PECAM-1)
CDH5	Cadherin 5 (VE-Cadherin)
circ3302	Circular RNA 3302
COL1A1	Collagen Type I Alpha 1 Chain
COL3	Collagen Type III
CTGF	Connective Tissue Growth Factor
d-flow	Disturbed Flow
DPP-4	Dipeptidyl Peptidase-4
EC	Endothelial Cell
ECM	Extracellular Matrix
eNOS	Endothelial Nitric Oxide Synthase
EndMT	Endothelial-to-Mesenchymal Transition
FGF	Fibroblast Growth Factor
FN1 (Fn1)	Fibronectin 1
FSP-1	Fibroblast-Specific Protein 1
HDL	High-Density Lipoprotein
HDAC	Histone Deacetylase
HSP47	Heat Shock Protein 47
HUVEC	Human Umbilical Vein Endothelial Cell
IA	Intracranial Aneurysm
IL	Interleukin
KD	Kawasaki Disease
Kdr	Kinase Insert Domain Receptor (VEGFR2)
KLF4	Krüppel-Like Factor 4
MMP	Matrix Metalloproteinase
MLKL	Mixed Lineage Kinase Domain-Like Protein
miRNA	MicroRNA
mTOR	Mammalian Target of Rapamycin
NF-κB	Nuclear Factor Kappa B
NO	Nitric Oxide
Notch	Notch Signaling Pathway
p-ERK1/2	Phosphorylated Extracellular Signal-Regulated Kinase 1/2
PDGF	Platelet-Derived Growth Factor
PECAM-1	Platelet Endothelial Cell Adhesion Molecule 1
PI3K	Phosphoinositide 3-Kinase
PPAR-γ	Peroxisome Proliferator-Activated Receptor Gamma
ROBO4	Roundabout Guidance Receptor 4
S100A4	S100 Calcium Binding Protein A4
scRNA-seq	Single-Cell RNA Sequencing
SMA (α-SMA)	Alpha Smooth Muscle Actin
SM22α	Smooth Muscle Protein 22 Alpha (TAGLN)
Smad	Mothers Against Decapentaplegic Homolog Protein
SNP	Single Nucleotide Polymorphism
SOX4	SRY-Box Transcription Factor 4
SOX18	SRY-Box Transcription Factor 18
SPARC	Secreted Protein Acidic and Rich in Cysteine
TAA	Thoracic Aortic Aneurysm
TAAD	Thoracic Aortic Aneurysm and Dissection
TAGLN	Transgelin
TGF-β	Transforming Growth Factor Beta
TGFBR2	Transforming Growth Factor Beta Receptor 2
Th17	T Helper 17 Cells
TNF-α	Tumor Necrosis Factor Alpha
VE-Cadherin	Vascular Endothelial Cadherin
Vim	Vimentin
vWF	von Willebrand Factor
Wnt	Wingless/Integrated Signaling Pathway
WNT11	Wnt Family Member 11
Zeb2	Zinc Finger E-Box Binding Homeobox 2
ZEB1/2	Zinc Finger E-Box Binding Homeobox 1 and 2
ZNF	Zinc Finger Protein
ZO-1	Zonula Occludens 1
